# Multiplexed Affinity-Based Separation of Proteins and Cells Using Inertial Microfluidics

**DOI:** 10.1038/srep23589

**Published:** 2016-03-30

**Authors:** Aniruddh Sarkar, Han Wei Hou, Alison. E. Mahan, Jongyoon Han, Galit Alter

**Affiliations:** 1 Ragon Institute of MGH, MIT and Harvard, Cambridge, MA 02139, USA; 2 Department of Electrical Engineering and Computer Sciences, Massachusetts Institute of Technology, Cambridge, MA 02139, USA; 3 Department of Biological Engineering, Massachusetts Institute of Technology, Cambridge, MA 02139, USA

## Abstract

Isolation of low abundance proteins or rare cells from complex mixtures, such as blood, is required for many diagnostic, therapeutic and research applications. Current affinity-based protein or cell separation methods use binary ‘bind-elute’ separations and are inefficient when applied to the isolation of multiple low-abundance proteins or cell types. We present a method for rapid and multiplexed, yet inexpensive, affinity-based isolation of both proteins and cells, using a size-coded mixture of multiple affinity-capture microbeads and an inertial microfluidic particle sorter device. In a single binding step, different targets–cells or proteins–bind to beads of different sizes, which are then sorted by flowing them through a spiral microfluidic channel. This technique performs continuous-flow, high throughput affinity-separation of milligram-scale protein samples or millions of cells in minutes after binding. We demonstrate the simultaneous isolation of multiple antibodies from serum and multiple cell types from peripheral blood mononuclear cells or whole blood. We use the technique to isolate low abundance antibodies specific to different HIV antigens and rare HIV-specific cells from blood obtained from HIV+ patients.

Isolation of specific proteins and cells from clinical samples that are complex, multi-component mixtures (for eg. blood) serves as the essential first step in analytical and preparative methods involved in a range of applications. For a large, clinically relevant class of low abundance target proteins and rare cells, such as antigen-specific antibodies or antigen-specific B and T cells, no easily accessible physical differences like size, density or charge exist, and binding affinity to a cognate antigen is the distinguishing characteristic that is used to isolate them before downstream molecular or cell-based assays, that require purified inputs, can be performed.

Current affinity purification methods for proteins and cells use binary separation of binding and non-binding fractions of the sample mixture. Isolation of multiple targets is performed serially using multiple binding, washing and elution steps using resins or magnetic beads coated with bait molecules^1^,^2^. This approach, while traditionally effective, is time consuming, low-throughput, and difficult to standardize and use for limited volume clinical samples due to the unavoidable loss and degradation of sample with repeated purifications. Multi-target magnetic cell separation has been proposed but demonstrated only for small bacterial cells using a microfluidic magnetophoresis device^3^ or for beads by sequential elution using specially designed, selectively displaceable DNA linkers^4^. Fluorescence activated cell-sorting (FACS) remains the standard method in multiplexed cell sorting but the high cost of instruments and technical expertise required makes this method relatively inaccessible. Also the manual handling steps in these methods or the nature of instrumentation (eg. jet-in-air formation in FACS) makes them challenging to apply to highly infectious clinical samples.

A multiplexed, yet inexpensive and high-throughput affinity separation method, applicable to proteins and cells, can accelerate the characterization of clinical samples in time-critical applications. For example, in the context of an infectious disease outbreak like the recent Ebola virus disease outbreak^5^ in West Africa, such a method can be used for the rapid isolation of antigen-specific antibodies and B cells or plasma cells harboring the most effective antibodies from rare resistant individuals or vaccinees. This can enable the development of novel diagnostic biomarkers^6^ and effective monoclonal therapeutics^7^,^8^ which can play a critical role in attenuating the outbreak.

Inertial microfluidics offers the advantage of high sample throughput in relatively inexpensive yet robust and easy-to-use devices, and can thus be adapted for use with a wide range of downstream assays. Earlier work using inertial microfluidic devices, which has been reviewed recently^9^, has demonstrated cell and particle focusing, isolation and analysis and has been widely applied to isolation of circulating tumor cells (CTC) in cancer. Commonly, these methods have used size, shape or deformability of particles and cells, which can directly affect their inertial focusing^10^. These are complementary to binding affinity for a cognate antigen or antibody. Also the use of inertial microfluidics for separation of molecules, in general, has been limited by their small size, which is usually way below the threshold of particle size above which inertial focusing is usable. Extension of inertial microfluidic separation techniques to affinity-based separation of molecules and cells can make it particularly well suited for use in the context of infectious diseases especially in resource-poor settings.

Here, we report an inertial microfluidic scheme for rapid and multiplexed affinity-based separation of proteins and cells, which is inexpensive, easy to automate and can work with small or large sample volumes. As shown in Fig. 1, this method involves a single binding step in which the sample is incubated with a mixture of microbeads of a number of different sizes each coated with a different capture agents (antigen or antibody). After binding, the mixture is flowed through a spiral microchannel device, which sorts the mixture into different outlets based on size. This device works on the principle of Dean Flow Fractionation (DFF)^11^. In DFF, particles above a certain size threshold when flowing through a spiral channel (d_p_/h > 0.07, where d_p_ is the effective particle diameter and h is the channel height) can be focused into distinct streams due to the superposition of size-dependent inertial lift forces (F_L_) and a drag force (F_D_) due to the Dean flow generated as a result of centrifugal acceleration of the fluid, indicated here by the counter-rotating fluid vortices it generates. The device height, particle sizes and outlet positions can be designed to match a single outlet to each stream containing target-bound beads of a single size (Outlets 2–4 in Fig. 1), which can thus be separated. All unbound beads and molecules or cells, below the focusing threshold size remain entrained in the Dean flow and can also be collectively guided into a separate outlet (Outlet 1 in Fig. 1). The output streams are collected and used for downstream processing of the isolated, bound targets either with or without elution from the beads. Here we develop and demonstrate this technique and use it in the context of HIV-specific antibody and cell isolation from blood obtained from HIV-positive patients.

## Results and Discussion

### Device Design and Characterization

**Figure 2:**
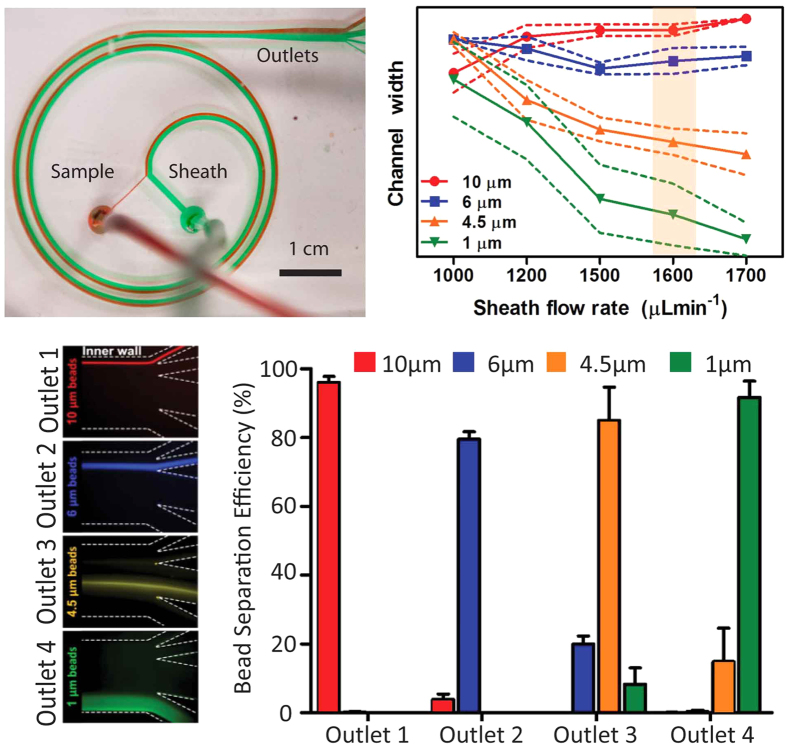
(**a**) Fabricated PDMS spiral microchannel device showing sample (red) and sheath (green) flows. (**b**) Locations of streams of beads of different diameters inside the device. Solid line indicates position of centre of stream while dotted lines indicate its edges. An optimized flow rate from the highlighted range was chosen for further experiments. (**c**) Focused bead streams flow in separate device outlets designed to capture them. (**d**) Bead separation efficiency as measured using flow cytometry.

A test spiral microchannel device as described above was fabricated (as shown in Fig. 2a, but with a single outlet) and suspensions of microbeads of various diameters were flowed through it along with a sheath flow to observe the focusing positions of the beads at different flow rates. After initial trials with a number of bead sizes, four bead sizes (d_1_ = 10 μm, d_2_ = 6 μm, d_3_ = 4.5 μm, d_4_ = 1 μm) were chosen which were the smallest set of sizes that could be reliably directed into distinct streams at a range of flow rates as shown in Fig. 2b. Outlets (n = 4) of the device for further use were then designed to capture these separate streams. The focusing of the streams of these beads of different sizes into the four outlets is shown in Fig. 2c. To verify bead separation, a mixed suspension of these beads was flowed into the device and the output from each outlet was collected. The input and the outputs were analyzed by flow cytometry and the separation efficiency of each bead size was verified as shown in Fig. 2d. Sample raw flow cytometry plots are shown in Figure S1. In general, the separation efficiency ranged from 80% to 95% and was repeatable (CV < 10%) between experiments. The difference in separation efficiency between bead sizes can be attributed to either the minor size variation in the input bead population or to imperfect outlet sizes in the current design, which can be further optimized. It is also worth noting here that beads smaller than those used in earlier work by others^12^ have been used here since lower bead sizes allow higher area per unit mass of beads and hence higher antibody binding per unit mass of solids in the suspension, which ultimately provides higher sample throughput in affinity separation.

### Antibody Separation

**Figure 3:**
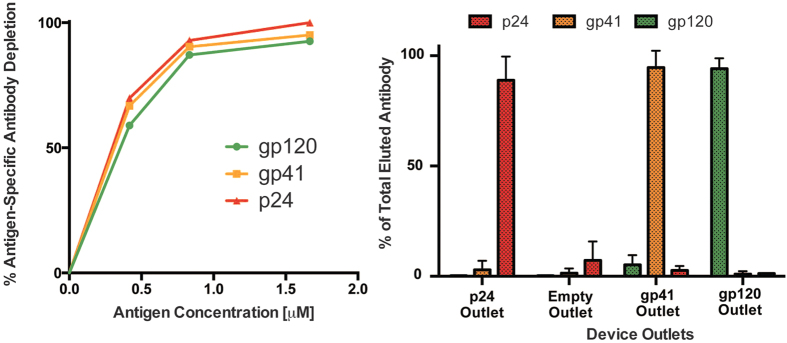
Multiplexed isolation of three different HIV antigen-specific antibodies. (**a**) Fraction of antigen-specific antibodies captured from samples after binding to a mixture of beads. Simultaneous capture of antibodies against three different antigens is observed. (**b**) Normalized antigen binding titer of antibodies eluted from beads obtained at each outlet. Pure antibodies specific to each antigen are obtained at the respective outlet to which the beads coated with that antigen were directed. Error bars represent standard deviation of three experiments.

We used the device designed above (h =  85 μm, n = 4) for multiplexed separation of antibodies specific to three different HIV antigens from serum obtained from HIV-infected patients as shown in Fig. 3. The three major HIV proteins p24, gp41 and gp120 were coated as baits on beads of three different sizes (10 μm, 4.5 μm and 1 μm beads respectively) via biotin-streptavidin linkage. The protein coating on beads was optimized for maximum coverage by varying the beads-to-protein mass ratio (Figure S2a). Both magnetic and non-magnetic polystyrene beads were tested. It was observed that magnetic beads had higher binding capacity for identical size (Figure S2b) and they were chosen for all further antibody separation experiments. Samples consisted of total Immunoglobulin G (IgG) fraction of serum (concentration of IgG at 1 mg/mL), which is known to contain <1% of antibodies specific to an antigen. In a single sample-binding step, mixtures of coated beads were incubated with samples (volume ~1 mL). Antigen-binding titers of the supernatant, after binding with the bead mixture and pelleting of the bead mixture, was measured using an Enzyme Linked Immunosorbent Assay (ELISA) to verify the simultaneous capture of antibodies against all three antigens. A bead concentration-dependent capture of all three antigen-specific antibodies was observed (as shown in Fig. 3a) and near-complete (~95%) capture was achieved. The antibody-bound beads were then re-suspended in sorting buffer and sorted using the device. Antibodies were then eluted from the beads obtained at each of the device outlets. Elution times were optimized by characterizing titers of various eluted fractions over time and an elution time of ~5 mins was found to be sufficient (Figure S3). The amount of HIV-specific antibodies in serum is known to vary between patients and cane be around 0.5–1% of total serum IgG^13^. A 1–5 μg amount of each antigen-specific antibody was isolated from 1 mg of total serum IgG using this technique. Overall, the milligram amount of input sample was processed using the device to isolate microgram amounts of target antigen-specific antibodies in less than 10 minutes after binding. This represents a more than 1000-fold improvement in microfluidic affinity separation throughput compared to earlier work^14^. Compared to the traditional macro-scale antibody separation technique based on repeated bind-elute steps this technique, which required almost a full day to separate three different antibodies, this technique presents a 10-fold reduction in time required as well as significant reduction in manual labor required.

The purity of eluted p24-, gp41- and gp120-specific antibodies obtained was verified using ELISAs for binding with each of the three antigens (as shown in Fig. 3b). Only minimal cross-contamination (<5%) was observed. Similar results were also obtained when isolating only two antigen-specificities (Figure S4). Eluted gp120-specific antibodies were then used in a functional assay for antibody-dependent cytotoxicity (ADCC). This assay creates an *in-vitro* model of antibody-induced lysis of virus-infected cells by recruiting natural killer (NK) immune cells. This involves both the antigen-binding (Fv) and the immune-activating (Fc) ends of antibody molecules. The results showed that the antigen-specific antibodies were enriched intact and verified that this functional property could be interrogated after separation (Figure S5). This further establishes the usefulness of the technique reported here as a multiplexed affinity-separation technique for antibodies which can be used for downstream functional assays.

### Cell Separation

We next tuned the bead sizes and device dimensions for multiplexed affinity-based cell separation. The eventual target cells of interest were antigen-specific cells, which are part of the peripheral blood mononuclear cells (PBMC) fraction, which consist of a mixture of a number of different cell types. Bead-binding of target cells was first tested by incubating freshly isolated human PBMC with 5-fold to 10-fold excess of beads of different sizes coated with antibodies against the CD3 surface marker protein, which is expressed on T cells. After binding, it was observed that dumbbell-like bound cell-bead pairs were formed (Figure S6). For bigger beads (6 μm, 10 μm and 15 μm) single bead-cell pairs were found. For smaller beads (1 μm and 2 μm), multiple beads were bound to single cells but all beads were found in a single cluster on one face of the cell. It was also observed that bead-cell binding was highly efficient when performed via an anti-fluorophore secondary antibody on the beads and a fluorophore-labeled primary antibody directed against the cell-surface marker (Figure S7). This scheme was then used for further experiments.

**Figure 4:**
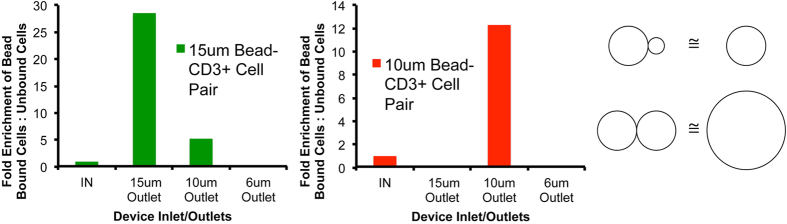
Optimization of bead sizes for affinity-based cell sorting. (**a**) Fold-enrichment of T Cells (~6–8 μm in diameter) bound to 15 μm and 10 μm beads into the 15 μm and 10 μm bead outlets respectively. (**b**) Bead-cell pairs show similar focusing positions as beads with the bigger of the two sizes or the sum of two sizes depending on if they are equal or unequal in size respectively.

**Figure 5:**
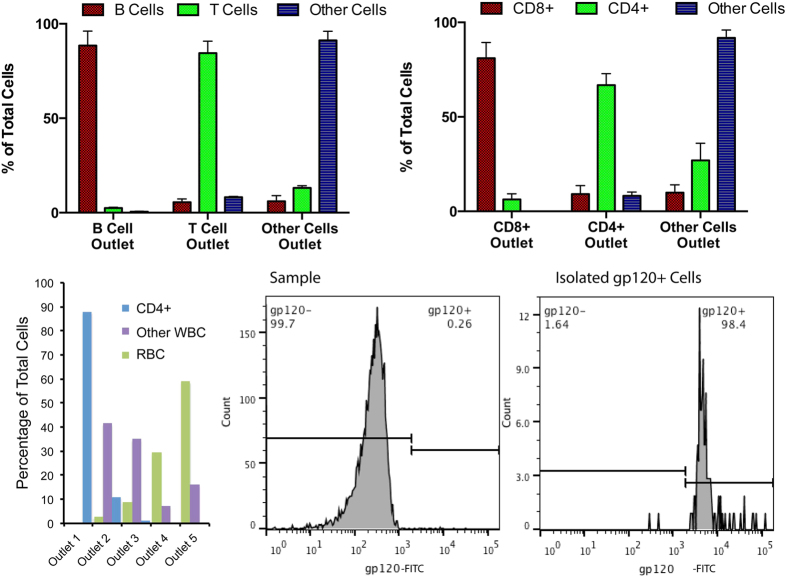
Demonstration of multiplexed cell sorting. (**a**) Separation of T Cells and B Cells from rest of PBMC (**b**) Separation of CD4+ and CD8+ cells from rest of PBMC. (**c**) Single-step isolation of CD4+ cells from whole blood. Error bars are standard deviation of three experiments. (**d**) Isolation or rare gp120-specific B cells from PBMC from HIV-infected donors.

Flowing the bead-bound mixture through the spiral microchannel device, it was observed that binding to beads with size greater than cell size resulted in a bead-size dependent deflection of bound cell-bead pairs away from the unbound cell stream. However binding to beads smaller than cell size resulted in no such deflection. This is shown in Fig. 4a (and Figure S8) by the enrichment of T cells (average diameter ~6–8 μm) bound to beads of various sizes coated with an anti-CD3 antibody. It was observed than when bound to 15 μm or 10 μm beads, T cells are focused and directed to the same device outlet as unbound 15 μm or 10 μm beads respectively. However binding to 6 μm beads also directs them to the 10 μm bead outlet only while binding to 1 μm beads results in no deflection and bound and unbound cells end up in the same outlet. Thus, we inferred that the focusing position in the device, of a bead-cell pair, was set by the bigger of the two or if beads and cells were of equal size, they had an additive effect on the focusing position (Fig. 4b). This can be compared to earlier observations^15^ of doublet particles in high-aspect ratio straight microchannels, where the additive effect was seen irrespective of the ratio of diameters of the two bound particles. This difference indicates an effect of particle shape on focusing positions in spiral microchannels, which needs to be further explored, possibly in future theoretical and computational investigations, for complete understanding and exploitation. However for the purposes of this work, based on these observations and inferences, 10 μm and 15 μm beads were chosen for all further cell-sorting experiments to drive the bound bead-cell pairs into their separate outlets away from unbound cells using a slightly different device design (h = 115 μm, n = 5) than from those used for antibodies. This enabled sorting of T and B cells (via anti-CD3 and anti-CD19 antibodies) and other unbound cells, from PBMC, into three different outlets as shown in Fig. 5a. The separation efficiency was tuned by varying the flow rate as show in Figure S9. Similarly CD4+ and CD8+ T Cells could be sorted using beads coated with antibodies against those surface markers as shown in Fig. 5b. Direct isolation of specific cells from whole blood was also performed as shown in Fig. 5c, which shows the isolation of CD4+ cells, while directing other white blood cells (WBC) and red blood cells (RBC) to other outlets of the device.

We then used this technique for the isolation of rare antigen-specific cells. For this, fluorophore-labeled antigen-tetramers were used to label the cells. Figure 5d shows the isolation of HIV gp120-specific B cells from PBMC from HIV patients. Only 0.26% of all B cells were gp120-specific, which amounts to only 0.025% of total cells in this sample. This rare fraction was isolated with >98% purity which represents a 4000-fold enrichment of the target cells. The isolated B cells were cultured and stimulating for antibody secretion. The antigen-specificity of secreted antibodies was then verified using a micro-engraving technique^16^ (Figure S8). This also shows that viable rare antigen-specific B cells could be rapidly and inexpensively isolated using this technique.

## Conclusions

In summary, we have demonstrated here a simple, flexible and highly extensible yet inexpensive scheme for high throughput, multiplexed affinity-based separation of both proteins and cells. It provides sufficient throughput in a single device (10^4^–10^7^ beads per second) to support a number of downstream applications. For protein separation, milligram-scale amounts could be processed and for cell separation, 1–5 million cells could be processed each in less than 10 minutes. Parallelizing multiple devices can further increase this throughput^17^. Multiplexing beyond the four bead sizes demonstrated here is also possible by further optimization of device geometry and bead sizes. The inertial focusing threshold (d_p_/h > 0.07) below which all beads flow into the outermost outlet sets the lower bound to bead sizes separable, and hence for the bead size useful for protein separation. For the cell-sorting scheme, the target cell size sets the lower bound for the useful bead size. Above these minimum required sizes, for both protein and cell separation, bead sizes need to different enough so that their focusing positions (say p_1_ and p_2_, set by the balance of inertial and Dean drag forces) and particle stream widths (say w_1_ and w_2_, set by dispersion including due to inherent variation in particle size) allow for baseline separation of particle streams in order to obtain good purity (p_1_ − p_2_ ≫ w_1_ + w_2_). Given the complex and coupled dependence of these parameters on the flow rate and channel and particle dimensions, it is difficult to speculate, without significant further theoretical or computational investigation, on the minimum size difference needed or the absolute maximum number of different particle sizes separable. A simple way to extend the number of sorted targets is by cascading devices tuned to different bead size ranges or by sequentially using this device along with other separation mechanisms such as magnetic sorting.

Ultimately, the use of a microfluidic platform for affinity separation also enables seamless integration of this sample preparation technique as a module into a complete lab-on-chip system, which would offer inexpensive, sample-to-answer automation and standardization. For example, microfluidic antigen-specific antibody isolation can be used to reduce the cost of purification in the monoclonal antibody manufacturing pipeline where up to 80% of the total cost is related to purification only^18^. It can also integrated with other techniques for example: microfluidic enzymatic digestion of antibody glycans^19^ and micro-capillary electrophoresis for glycan sequencing^20^ to develop an integrated antigen-specific antibody glycosylation analysis^13^ chip. This can enable rapid discovery of novel glycan biomarkers, which are an upcoming class of useful biomarkers for a range of diseases. Similarly, rapid isolation of antigen-specific B cells integrated with microfluidic single B cell sequencing^21^ and potentially antibody expression can enable rapid identification of novel antibodies which currently is a laborious process that requires specialized FACS machines that can handle highly infectious samples. Especially in the context of emerging epidemics in resource-poor settings, where monoclonal therapeutics are needed rapidly, this can offer critically needed acceleration to the antibody discovery and development process. An integrated, rapid CD4 counter^22^ or a CTC separator^23^, directly from whole blood can also be developed using this technique as the front-end cell separator module. Overall, due to its relative simplicity and robustness, we envision this technique to be useful both as a standalone sample preparation technique as well as for use in integrated lab-on-chip systems, both in the context of infectious diseases and beyond.

## Materials and Methods

### Device Fabrication and Operation

The microfluidic device was fabricated using a standard soft lithography approach. Briefly, the spiral microchannel pattern drawn using AutoCAD (Autodesk) and transferred to a dark-field chrome mask (Fineline Imaging) was used for photolithography to create a 10 μm thick AZ4620 photoresist pattern on a standard 6-inch silicon wafer which then underwent Deep Reactive Ion Etching to create the channel pattern whose depth (h) was controlled via the etching time. Note that the specific device dimensions, namely channel height (h) and number of outlets (n) are designed for each application separately by experimental optimization. Specifically, two designs of (h =  85 μm, n = 4) and (h = 115 μm, n = 5) were used for antibody and cell separation respectively while the radius and length of the spiral are as described in earlier work^11^. The patterned silicon wafer was silanized with trichloro(1H,1H,2H,2H-perfluorooctyl) silane (Sigma Aldrich) before PDMS prepolymer mixed 10:1 with curing agent was poured on it and baked at 95 C for at least 1 hr. The cured PDMS was then peeled from the silicon wafer and used as a mold for PDMS recasting resulting in the final PDMS device. Holes were punched for fluidic inlets and outlets using a biopsy punch (1.5 mm) and the PDMS device was irreversibly bonded to a 75 × 50 mm glass slide by exposure to air plasma followed by assembly and baking at 95 C for at least 1 hr.

The device was mounted on an epifluorescence-equipped inverted microscope (Olympus IX71) stage. Sample bead mixtures suspended in sorting buffer (1XPBS, 0.1%BSA, 1 mM EDTA) and sheath sorting buffer flows were introduced into the device using Tygon tubing (OD: 1.5 mm, ID: 1 mm) connected to plastic syringes driven by syringe pumps (Harvard Apparatus, PHD2000). The flow rate ratio between sample and sheath flow was maintained at 1:10 to form a sample stream at the outer wall, which could be visualized by using fluorescent microparticles. During device characterization, fluorescent microparticles of various sizes were used and the focused stream positions were recorded as images or videos using a CCD camera (PCO Imaging Sensicam QE) and μManager software (http://www.micro-manager.org). For bead sorting, initially a sample consisting of a suspension of 1μm fluorescent beads in sorting buffer was introduced and the position of the input and output sample stream at the outer wall was verified and flow rates were adjusted if necessary to achieve this. For majority of devices, no such tuning was needed. However for a few devices, up to 2–3% change in sheath flow rate compensated for any device-to-device variation. Then sample bead mixtures were introduced and collected via tubing connected to device outlets into tubes of appropriate volume.

### Samples

Whole blood from healthy donors was obtained from Research Blood Components Inc. Serum and PBMC from HIV-infected individuals were obtained from Ragon Institute cohorts. The study was approved by the Massachusetts General Hospital Institutional Review Board and each subject gave written informed consent. The methods were carried out in accordance with the approved guidelines and regulations. Note that appropriate biosafety guidelines need to be followed for the handling of infectious specimens.

### Antibody Separation

#### Microbead Preparation

Antigens: gp120 (YU2, IT-001-0027p, Immune Technologies), gp41 (HXBC2, IT-001-005p) and p24 (HXBc2, IT-001-017p, Immune Technologies), were biotinylated using a long-chain NHS-ester activated biotinylation reagent (EZ-Link NHS-LC-LC Biotin, Pierce) and dialysed overnight into 1XPBS to remove excess biotin. Streptavidin-coated polystyrene (Bangs Labs) or magnetic polystyrene (Microparticle Gmbh) beads were washed and incubated for 1 hr at 4 °C with different bead: antigen ratios. A saturating ratio was arrived at by measuring amounts of antigen depleted using a Micro BCA Protein Assay (Pierce) and then used for all further experiments (Figure S2). Antigen-coated beads were washed three times with 1xPBS and were mixed at a ratio set to obtain equimolar amounts of all antigens in the mixture. Effective antigen concentration in the bead suspension could be calculated from these molar amounts and bead suspension volume.

#### Sample Binding

Antibodies (IgG) were separated from serum samples from HIV infected subjects using Melon Gel (Thermo Scientific) according to manufacturer’s instructions and resuspended at a concentration of 1 mg/ml. The coated bead mixture was incubated with this sample for at least 3 hrs at 4 ^o^C at different effective antigen concentrations to study antigen-specific antibody depletion. After binding, the beads were pelleted either by centrifugation or using a magnet and the bepleted sample was removed before washing and resuspension of beads in sorting buffer at an effective bead concentration of ~1 × 10^6^/ml.

#### Antibody Elution

Sorted beads collected at the device outlets were each pelleted and exposed to elution buffer for different amounts of time followed by pelleting of beads and removal and neutralization of elute. The details of various elution buffers tested are presented with results in Figure S3.

#### Antibody Characterization

Antigen-binding titers of eluted antibodies was determined by ELISA according to previously established protocols^6^. Briefly, each antigen was coated on separate 96-well polystyrene plates, and samples were allowed to bind to all, followed by binding with HRP-conjugated anti-human IgG. Colorimetric substrate was added and the plates were read for absorbance after quenching the conversion. Antibody Dependent Cellular Cytotoxicity (ADCC) activity of antibodies was characterized using a rapid fluorometric ADCC (RFADCC) assay with NK cells as effectors, as previously described^24^. Briefly, percentage loss of a viability dye from double-stained gp120-coated target cells in presence of a 10-fold excess of effector cells and an antibody concentration of 50 μg/ml was measured. HIV-IG (NIH AIDS Reagent Program, Catalog#3957) and pooled IgG from HIV negative donors were used as positive and negative controls respectively.

### Cell Separation

#### Samples

Target cells were isolated either from Peripheral Blood Mononuclear Fraction (PBMC) or whole blood as starting sample. For healthy donors, PBMC were isolated from fresh whole blood obtained in Acid Citrate Dextrose (ACD)-coated tubes using density gradient centrifugations with Ficoll-Hypaque (Sigma Aldrich Inc) as per manufacturers recommendations. For HIV-infected donors, frozen PBMC was obtained from the Ragon Institute central sample repository and used after washing away added cryoprotectants.

#### Antibodies, Microbead Preparation and Sample Binding

Streptavidin-coated polystyrene beads were incubated with biotinylated antibodies obtained from vendors specified below. Antibody-coated beads were then incubated with the cell mixtures while undergoing slow tumbling at room temperature for 20 minutes. Two modes of binding were initially tested – direct binding of unlabeled sample cell mixtures with beads coated with anti-target antibodies (for eg. PBMC + anti-CD3-15 μm beads for T cell separation) or binding of fluorophore labeled samples with beads coated with anti-fluorophore antibodies (for eg. PBMC labeled with Fluorescein Isothiocyanate (FITC)-antiCD3 + anti-FITC-15 μm beads for T cell separation). The latter mode was found to provide better binding efficiency as shown in Figure S7 and was used for all further cell sorting experiments. For multiplexed cell sorting two different fluorophore-labeled antibodies and anti-fluorophore antibodies were used (for eg. PBMC labeled with FITC-anti-CD3 and PE-anti-CD19 + anti-FITC-15 μm beads + anti-PE-10 μm beads for T cell and B cell separation).

For rare gp120-specific cell sorting, PBMC were labeled with a Phycoerythrin (PE) labeled gp120 tetramer that was prepared by incubating biotinylated gp120 (prepared as above) with 1/4^th^ the molar amount of Streptavidin-PE. Samples were incubated on ice for 20 minutes with fluorophore-labeled antibodies or fluorophore-labeled tetramers and washed thrice at 4 C with 1XPBS. Labeled samples were incubated at room temperature with anti-fluorophore antibody-coated bead mixtures for 30 minutes with slow tumbling. Biotinylated, FITC-labeled or PE-labeled anti-CD3, anti-CD4, and anti-CD19 were obtained from Abcam Inc. Biotin-anti-PE and Biotin-anti-FITC were obtained from Biolegend Inc.

#### Flow cytometry

Cells were characterized before and after binding with beads and after sorting using the device using a BD Accuri C6 flow cytometer. Flow cytometry gates to identify various antibody-labeled cells were set as per standard instrument protocols using single-labeled and unlabeled controls. Also control samples with and without capture beads of each of the two sizes were used to set the bead-bound cells gates. This is shown for an example case of CD4+ and CD8+ cells in Figure S11. These gates were then used to count the unbound and bound specific cells in all samples before and after sorting.

#### Antibody secretion assay

PBMC (~1 × 10^6^ cells total) and gp120-specific cells after isolations using the devices were cultured for 7 days in appropriate media supplemented with cytokines (IMDM Glutamax^TM^, 10% FBS, CD40L: 50 ng/mL, IL-2: 10 ng/mL, IL-21: 50 ng/mL, all from Life Technologies) for stimulation of antibody production as per protocols from Huang *et al.*^25^. After 5 days, cells were removed from culture, washed in 1XPBS and used for microengraving as per protocols from Love *et al.*^16^. In summary, cells were loaded in complete medium at roughly single-cell per well or lesser density in 50 μm × 50 μm50 μm nanowell arrays bonded on standard microscope glass slides. The well arrays were then sealed in a hybridization chamber with another microscope slide coated with capture antibodies and the stack was incubated for 2 hours at 37 C. The capture slide was then removed, washed and labeled with two secondary labels: FITC-labeled Anti-Human IgG and PE-labeled gp120 tetramers. The slide was then imaged using a fluorescence microscope for fluorescent spots that resulted due to the secretion of antibodies from the stimulated cells. The percentage of antigen-specific cells was estimated from the percentage of spots that showed both green and red fluorescence (FITC+, PE+ or IgG+, gp120+) versus those that were only green fluorescent (FITC+, PE− or IgG+, gp120−) as shown in Figure S10.

## Additional Information

**How to cite this article**: Sarkar, A. *et al.* Multiplexed Affinity-Based Separation of Proteins and Cells Using Inertial Microfluidics. *Sci. Rep.* **6**, 23589; doi: 10.1038/srep23589 (2016).

## Supplementary Material

Supplementary Information

## Figures and Tables

**Figure 1:**
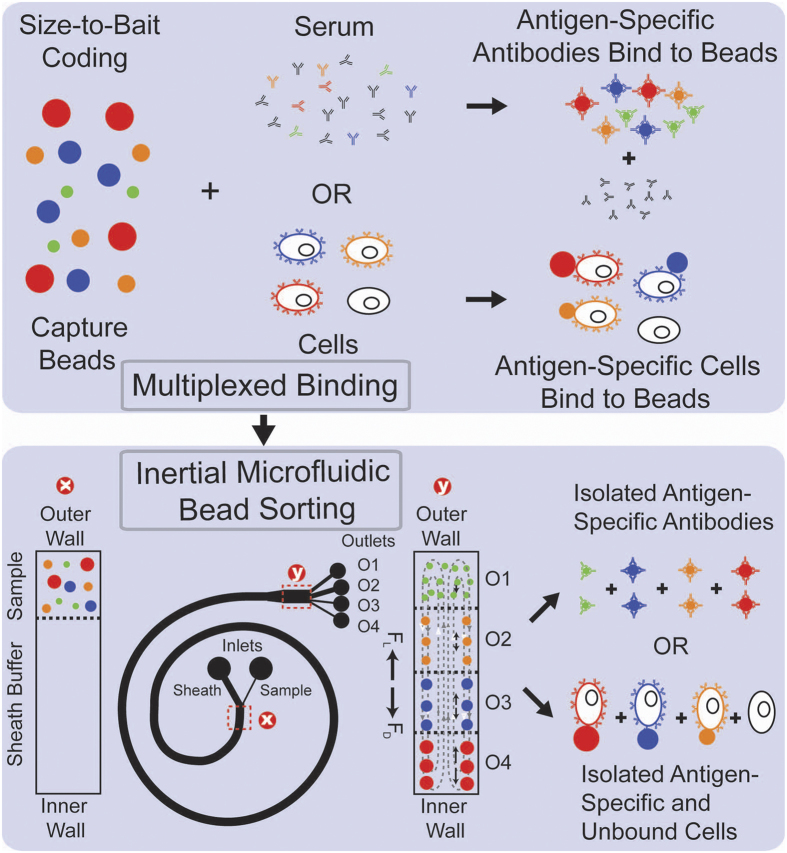
Principle of multiplexed protein and cell sorting using a Dean Flow Fractionation (DFF) device. Beads of different sizes coated with different capture agents bind in a single step to the corresponding different antibodies or cells and are then sorted based on their size using the DFF device after which they can be post-processed for downstream analysis.
