# Supplementary material for: Multiplexed Affinity-Based Separation of Proteins and Cells Using Inertial Microfluidics
Source: Sci Rep. 2016 Mar 30;6:23589. doi: 10.1038/srep23589 (PMC4812309; doi:10.1038/srep23589)

Supporting Information

Multiplexed Affinity-Based Separation of Proteins and Cells Using Inertial Microfluidics

Aniruddh Sarkar,[†, ‡] Han Wei Hou[‡], Alison E. Mahan,[†] Jongyoon Han[‡, §] and Galit Alter[†]

[†]Ragon Institute of Massachusetts General Hospital, Massachusetts Institute of Technology and Harvard

[‡]Department of Electrical Engineering and Computer Science, [§]Department of Biological Engineering, Massachusetts Institute of Technology, Cambridge, MA 02139, United States

Figure S1. Flow cytometric evaluation of input mixture of beads and separated output streams.

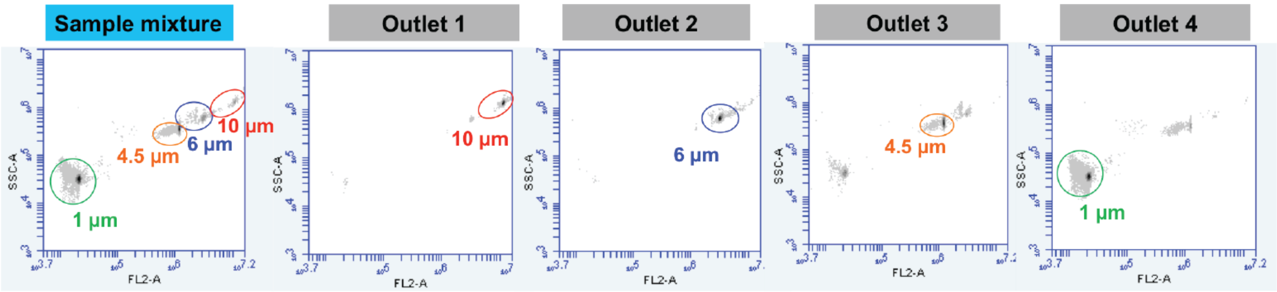

Figure S2. Optimization of bead to antigen coupling. a) Depletion of biotinylated antigen, measured using a bicinchoninic acid assay (BCA) for total protein, after binding of the same amount of starting antigen to different amounts of beads. Bead concentration is normalized to a nominal bead concentration arrived at from its reported biotin binding capacity. b) Biotin-binding capacity of polystyrene and magnetic polystyrene beads of three different sizes.

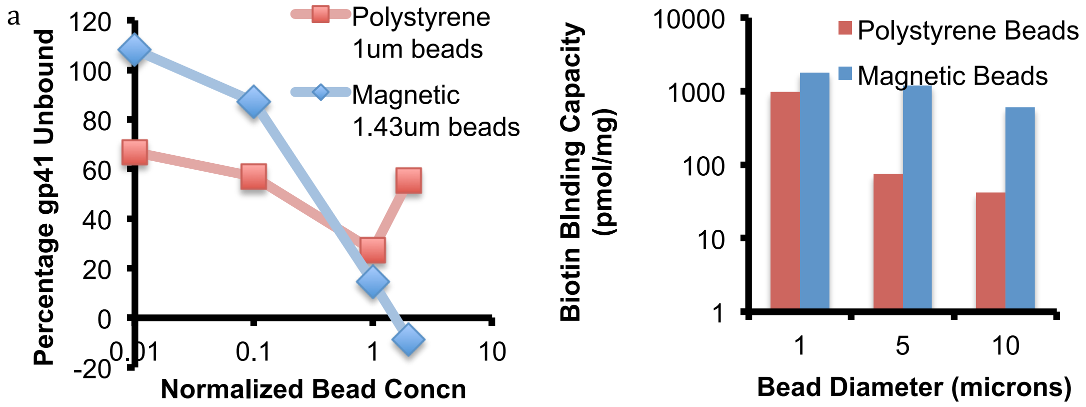

Figure S3. Optimization of antibody elution from beads. Binding titer obtained from a gp41-binding ELISA of antibodies eluted from identical amounts of gp41-coated beads after incubation with antibody sample and elution under different conditions.

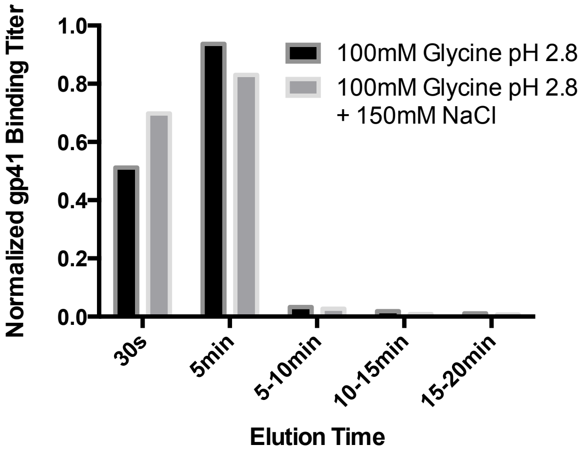

Figure S4. Multiplexed isolation of two different antigen-specific antibodies. Normalized antigen binding titers of antibodies eluted from beads obtained at each outlet. Pure antibodies specific to each antigen are obtained at the respective outlet to which the beads coated with that antigen were directed.

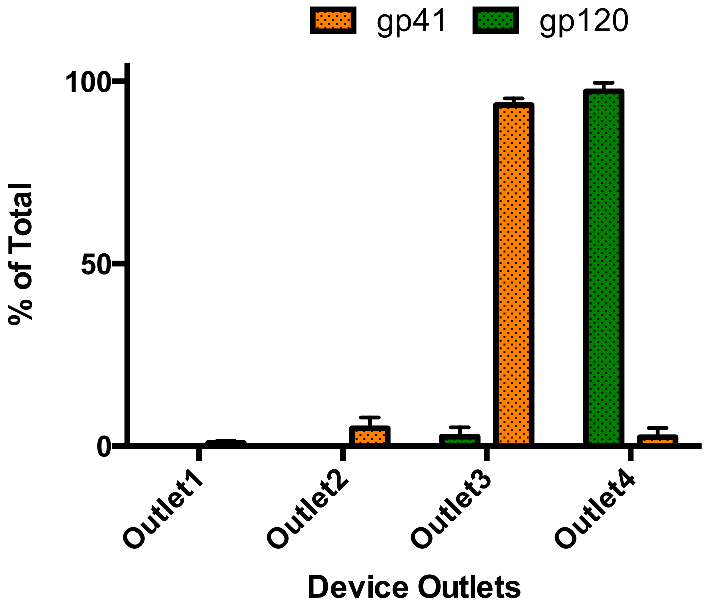

Figure S5. Antibody-Dependent Cellular Cytotoxicity Assay. Percentage loss of viability indicating dye (CFSE) from a population of gp120-coated target CEM cells in presence of a 10-fold excess of freshly isolated NK cells as effector cells and antibodies from various samples tested. An undepleted antibody sample from an HIV-infected subject shows cytotoxicity. Incubation of this sample with gp120-coated microbeads at increasing effective gp120 concentration causes decrease of this function. Antibodies eluted from these microbeads retain function. HIVIG and HIV negative IgG are used as positive and negative controls respectively.

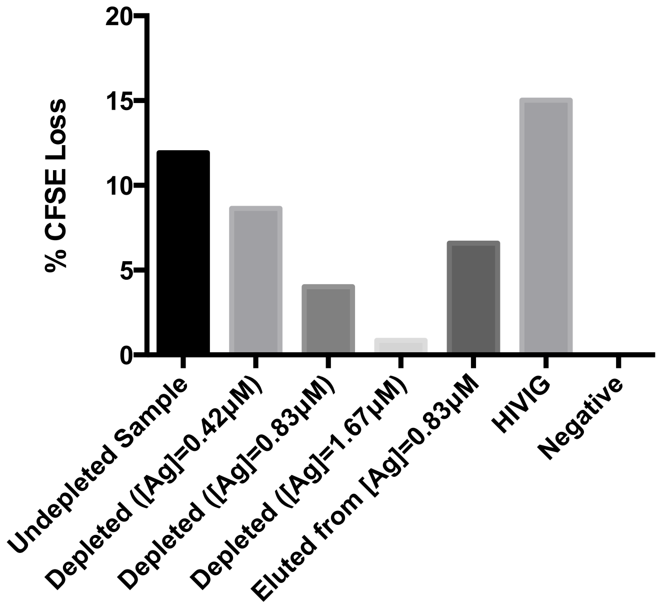

Figure S6. Cells bound to antibody-coated microbeads of various sizes. Cells were coated with phycoerythrin(PE)-labeled anti-CD3 antibody and beads of different sizes were coated with anti-PE antibody. Dumb-bell shaped single cell-bead pairs are observed for bigger beads while for smaller beads multiple beads bind to the cell but cluster to one side to form an overall dumb-bell shape.

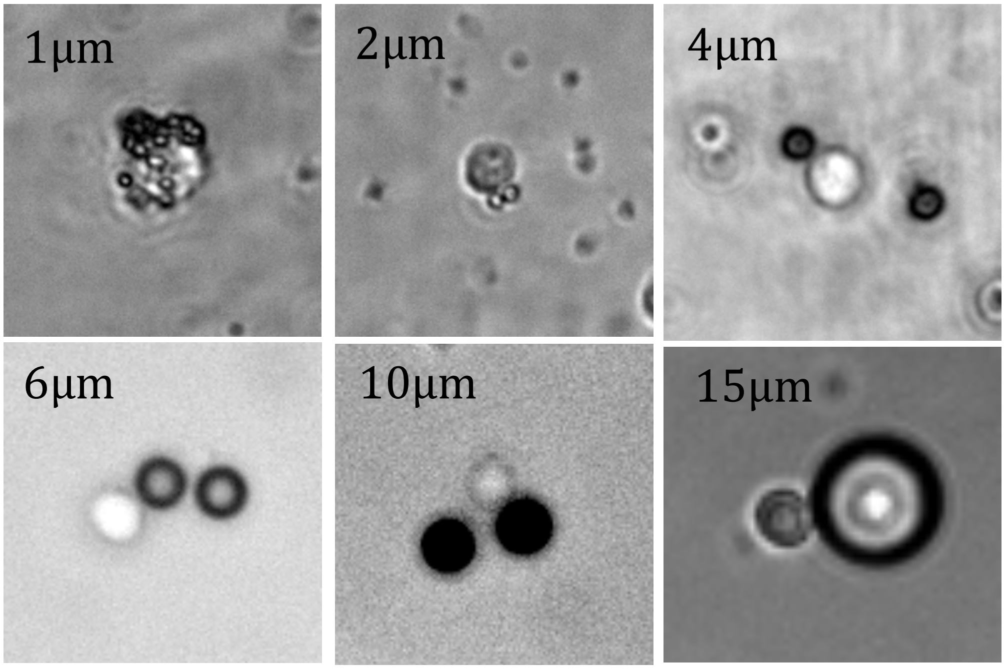

Figure S7. Optimization of bead-cell binding. Comparison of bead-cell binding using anti-CD3 antibody directly on 15μm beads or using a fluorophore-tagged anti-CD3 antibody on the cell and an anti-fluorophore antibody on the 15μm bead.

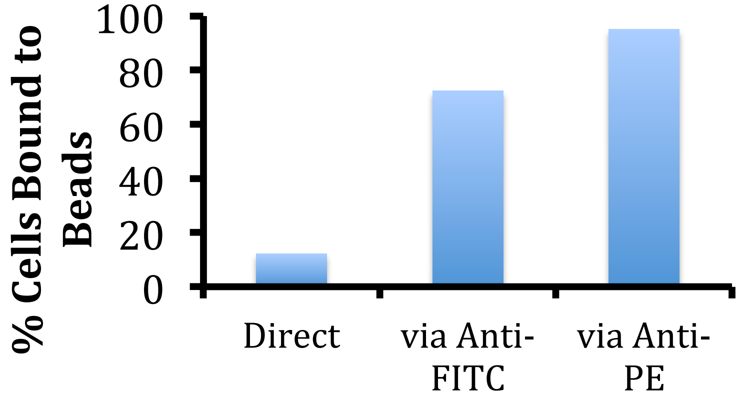

Figure S8. T Cells (~6-8μm in diameter) bound to 6μm beads are enriched into the outlets O2 (10μm) outlet while no enrichment is seen for T Cells bound to 1μm beads.

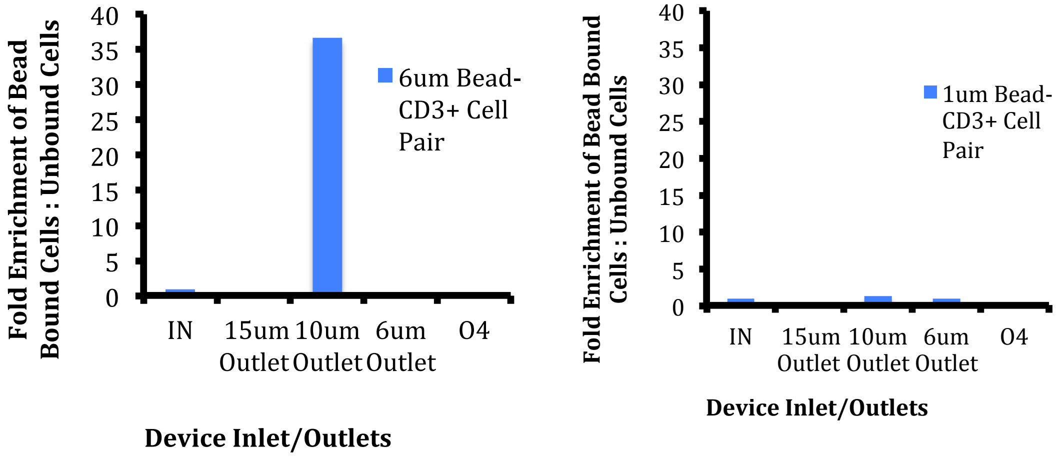

Figure S9. a-d) Flow rate optimization for separation of T Cells and B Cells from rest of PBMC by binding to 10μm anti-CD3 coated bead and 15μm anti-CD19 coated beads. e,f) Separation of CD4+ and CD8+ cells from rest of PBMC also at flow rate as optimized above. The conditions providing best sorting efficiency among those tested are outlined in red boxes.

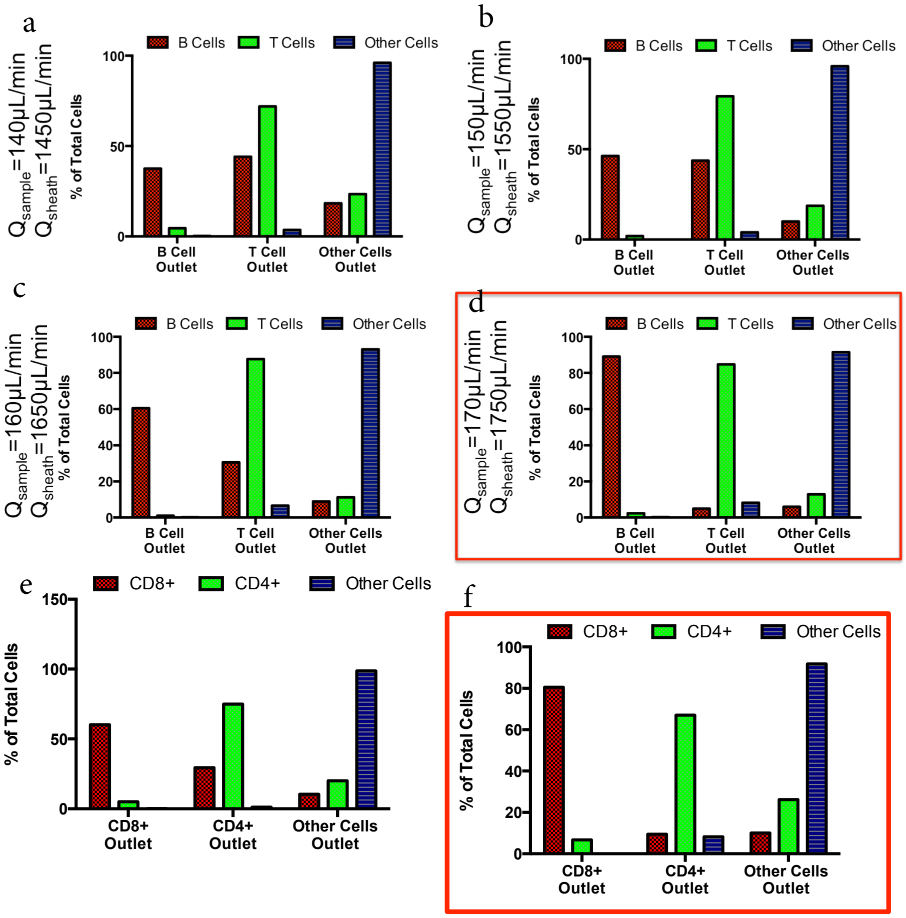

Figure S10. Antigen-specificity of antibodies secreted by single sorted cells was verified by microengraving after culturing the sorted cells and stimulating them to secrete antibodies. a) Fluorescence micrograph of capture slide after dual secondary labeling with FITC-anti-Human IgG and PE-gp120 tetramer. b) Counts of IgG+ and gp120+ spots

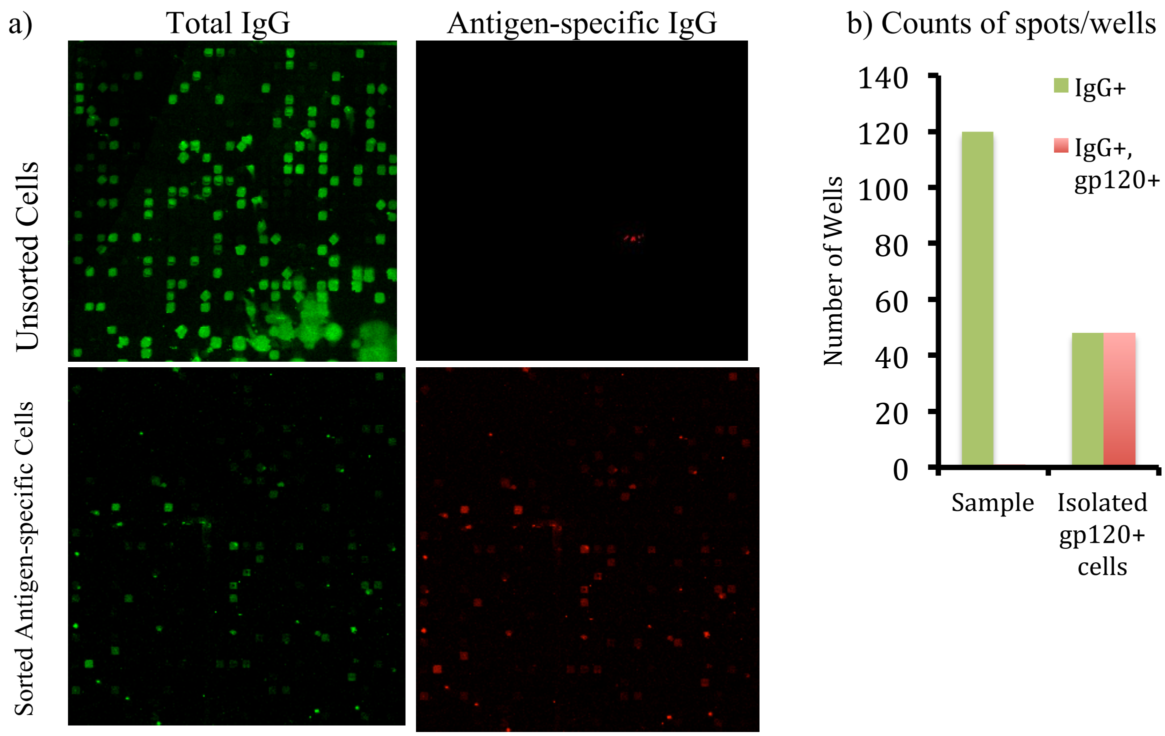

Figure S11. Flow cytometry of unlabeled, antibody-labeled and bead-bound cells to set gates to characterized cells before and after sorting. Arrows indicate events gated and plotted for a different set of parameters. a) Lymphocyte gates for PBMC are set using forward and side scatters. b) PBMC labeled with FITC-anti-CD4 antibody only. c) PBMC labeled with PE-antiCD8 antibody only. d,e,f) PBMC labeled with FITC-anti-CD4 and bound with 10μm beads coated with anti-FITC antibody. g,h,i) PBMC labeled with PE-anti-CD8 and bound with 15μm beads coated with anti-PE antibody. j) PBMC labeled with both FITC-anti-CD4 and PE-anti-CD8 and k,l) bound with both 10μm beads coated with anti-FITC antibody and 15μm beads coated with anti-PE antibody.

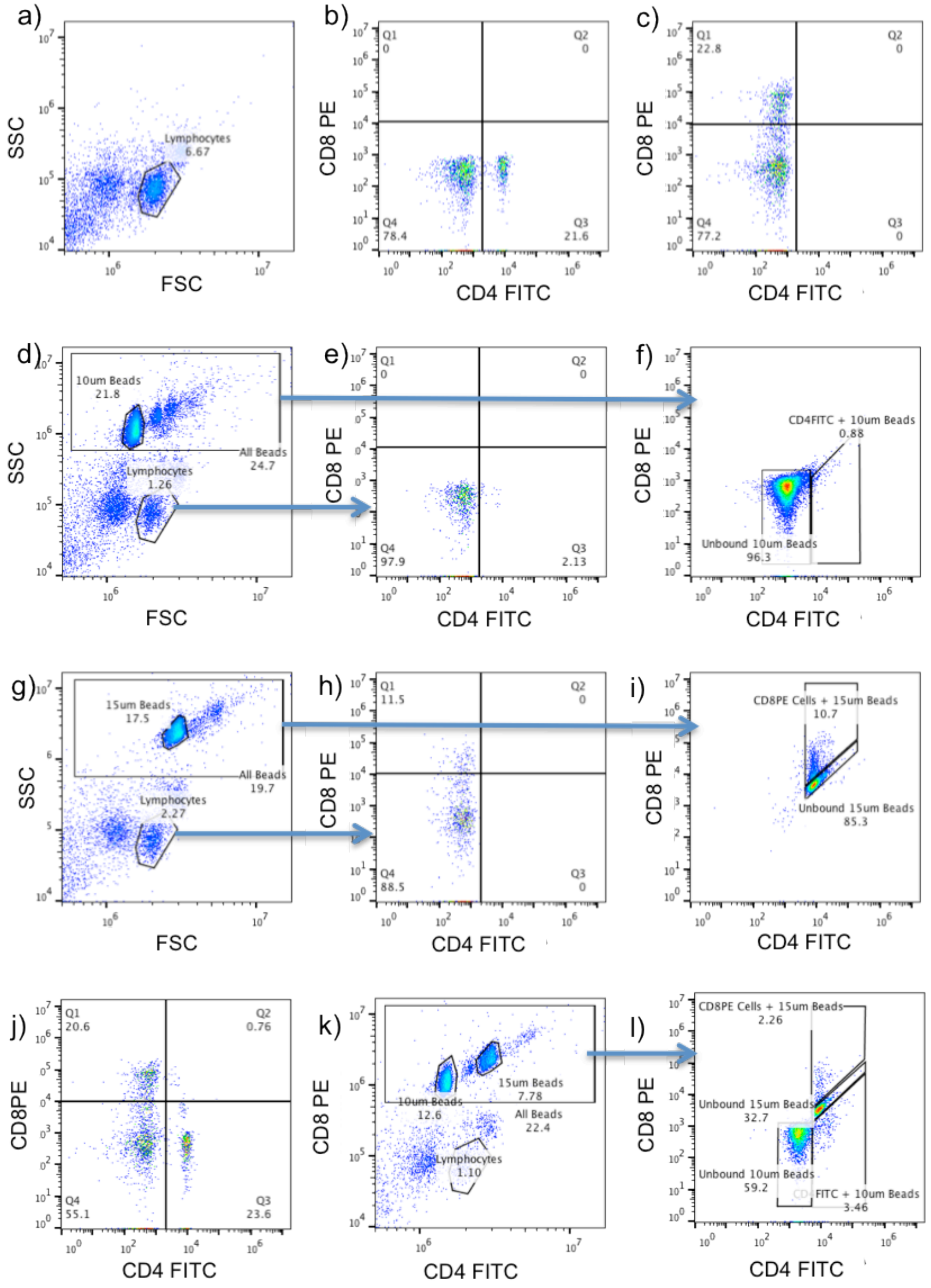

Figure S12. Flow cytometry of sorted cells obtained at the device outlets after flowing sample in Fig. S11 (k,l) above through the device. a,b) Cells obtained at Outlet 1 (15µm Outlet) and c,d) Outlet 2 (10µm Outlet).

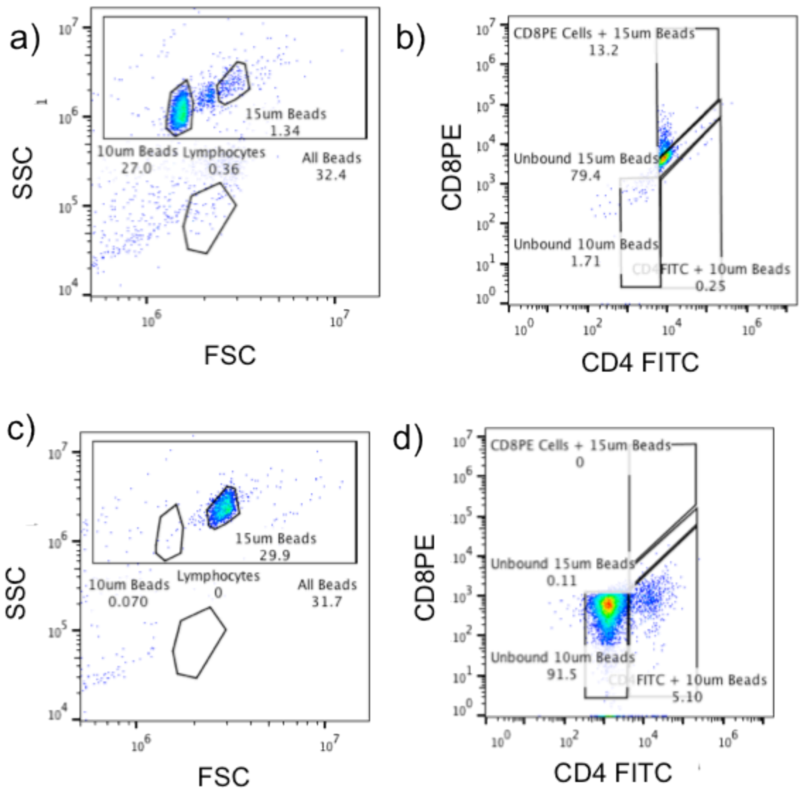

Supplement: Supplementary Information [file srep23589-s1.pdf]
